# Observation of Voriconazole in the Treatment of Liver Failure Complicated With Invasive Pulmonary Fungal Infection Induced by Chinese Patent Medicine in Teenagers: 2 Case Reports

**DOI:** 10.3389/fphar.2022.862222

**Published:** 2022-04-20

**Authors:** Qian Su, Jinjin Pan, Li Zhang, Lingling Xia, Yufeng Gao, Jiabin Li

**Affiliations:** Department of Infectious Diseases, The First Affiliated Hospital of Anhui Medical University, Hefei, China

**Keywords:** Chinese patent medicine, drug-induced liver failure, Invasive Pulmonary Fungal Infections (IPFI), autoimmune-like phenomena, voriconazole (VCZ), case report

## Abstract

**Background:** Drug-induced liver injury (DILI) caused by Chinese patent medicines is increasing in China. The incidence of invasive fungal infections (IFIs) is increasing due to the suppression of the immune function in greater numbers of patients. Invasive procedures such as deep vein catheterization and the use of glucocorticoids are also predisposing factors to IFIs. The clinical presentation of IFI in teenagers is often atypical, challenging to diagnose, difficult to treat, and associated with a high fatality rate.

**Case presentation:** Herein, we report 2 teenagers with liver failure after receiving oral Chinese patent medicines. Case 1 was a 14-year-old boy who presented with subacute liver failure who had been administered a Chinese patent medicine that included acetaminophen. Administration of glucocorticoids and non-bioartificial liver treatment improved his condition. Subsequently, invasive pulmonary *Aspergillus* (IPA) was diagnosed and was successfully treated with voriconazole for 85 days. Case 2 was a 17-year-old girl who presented with acute liver failure after taking the Chinese patent medicine QubaiBabuqi tablets for vitiligo. Chest computed tomography (CT) revealed multiple pulmonary nodules with an intermittent low-grade fever, and she was diagnosed with IPA. She was initially treated with caspofungin (23 days) and then voriconazole (406 days) for 429 days. Her liver function returned to normal, and lung lesions were absorbed in 2 patients. At the same time, two to three histopathological examinations of the liver biopsy showed that the drug-induced autoimmune-like phenomena could be improved by glucocorticoid therapy.

**Conclusion:** To the best of our knowledge, this is the first report of the successful treatment of 2 cases of liver failure (Child–Pugh class C) caused by Chinese patent medicines complicated with IPA in teenagers. Drug-induced autoimmune-like phenomena could be improved by glucocorticoid therapy.

## Introduction

Drug-induced liver injury (DILI) refers to liver hepatotoxic injury caused by a drug itself or its metabolites, and can be categorized as hepatocyte, cholestatic, and mixed types. DILI accounts for <1% of the cases of acute liver injury seen by gastroenterologists but is the most common cause of acute liver injury in the United States and Europe (Kullak-Ublick et al., 2017). Immune mechanisms are an important part of the pathogenesis of DILI and may manifest as DILI with an autoimmune-like phenomenon with a portion of patients developing drug-induced autoimmune hepatitis (European Association, and for the Study of the liver, 2015). These 2 conditions have similar clinical symptoms, and accurate diagnosis is challenging. After searching the PubMed database, there is no similar report at home and abroad.

Due to the physiologic and anatomic characteristics of teenagers, drug clearance via the kidneys, liver, and lungs and drug biotransformation are poor; thus, many drugs can cause liver damage. In addition, teenagers are more prone to mitochondrial dysfunction due to drugs, increasing their susceptibility to severe liver injury or acute liver failure (ALF). Liver failure can be caused by many factors, and with severe liver damage, the only curative treatment is liver transplantation. Infection is a common complication in patients with liver failure, the frequency of liver failure complicated with an invasive fungal infection (IFI) was 2–15%, with the main pathogenic agents *Candida albicans* and *Aspergillus* and main primary sites the lungs and oral cavity (Schmiedel and Zimmerli, 2016). The mortality rate of patients with liver failure is extremely high, and the risk of death is further increased when an IFI is present.

Herein, we present 2 case reports of pediatric liver failure (Child–Pugh class C) with drug-induced liver failure accompanied by autoimmune-like phenomena and complicated by an IFI due to Chinese patent medicines for the first time.

## Case Presentation

### Case 1

A 14-year-old boy (weight: 90 kg; height: 175 cm; BMI: 29.39 kg/m^2^) was admitted to our hospital on 22 June 2020 with symptoms of yellow eyes and diarrhea for more than 20 days. The child had been treated with a Chinese patent cold medicine (that included acetaminophen) for an upper respiratory tract infection 30 days prior. He had never taken the medicine before, and during treatment, a maculopapular rash developed on the trunk. The local hospital’s laboratory studies on June 4 showed a total bilirubin (TBil) of 277 μmol/L, direct bilirubin (DBIL) of 150.2 μmol/L, alanine aminotransferase (ALT) of 580 U/L, and aspartic acid aminotransferase (AST) of 320 U/L. On June 14, he was treated with methylprednisolone 40 mg/d intravenously for 3 days, and the rash improved. Liver function was noted to be aggravated 1 week later, and he was treated with prednisone acetate 20 mg orally every day from 22 June 2020, but his condition did not improve.

He was allergic to penicillin, and there was no history of any significant disease in his family. Moderate jaundice of the skin and sclera was noted, and no cardiopulmonary abnormalities were found.

**FIGURE 1:**
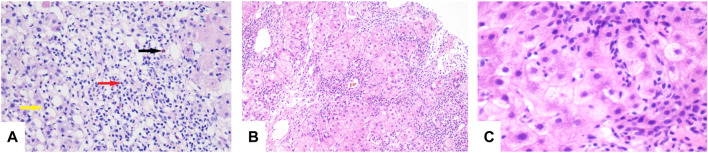
**(A)** Lymphocytes (red arrow), eosinophils (black arrow), and neutrophils (yellow arrow) seen in the portal duct area (HE, original magnification × 200). **(B)** “Rose garland” formation (HE, original magnification × 100). **(C)** Lymphocytes “penetrate” (HE, original magnification × 600).

A liver biopsy was performed on June 24, and pathological examinations were consistent with DILI (G3 S1) (Figure 1A). Pathological sections were sent to Renji Hospital, School of Medicine, Shanghai Jiao Tong University, for consultation, and after review, the diagnosis was autoimmune hepatitis (acute-severe). Mycophenolate mofetil (MMF) 750 mg/d (250 mg t.i.d.) orally began on 3 July 2020, and after 4 days, his TBil, PT, and INR levels had increased to 250.30 μmol/L, 23.2 s, and 2.12, respectively. Based on the laboratory studies and his condition, he had progressed to subacute liver failure (SALF). He was placed on the liver transplant list, and a complete pre-liver transplantation workup was performed.

Prednisone acetate was changed to methylprednisolone 30 mg q12 h ivgtt on 9 July 2020, and hepatic encephalopathy was diagnosed on July 10. MMF was discontinued on July 11, and he was given intravenous gamma globulin 20 g/d, but there was no improvement in his condition after 5 days of treatment. He was given treatment with a non-bioartificial liver (NBAL) starting on July 21, and after 5 treatments (from July 21 to August 3, once every 3–4 days) in combination with integrative medical treatments including liver protection, plasma infusion, and human albumin, his symptoms improved.

**FIGURE 2:**
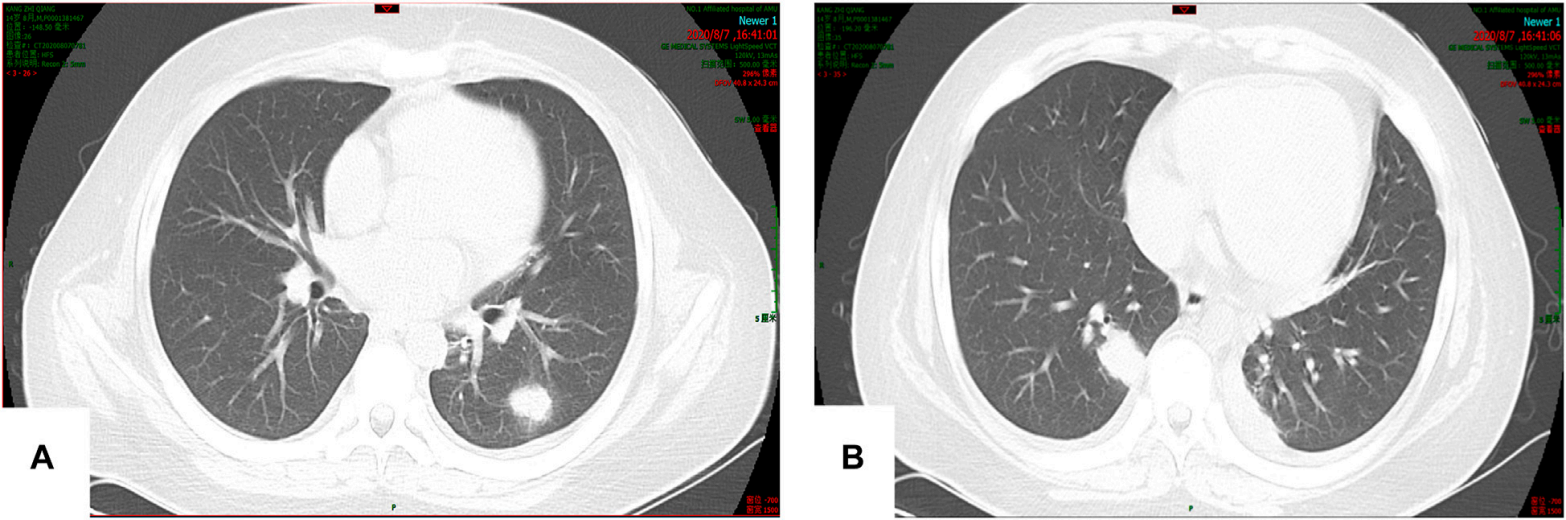
**(A,B)** Multiple nodular soft tissue shadows were observed in both lungs. The larger one was located in the lower lobe of the right lung (15 × 23 mm).

On 7 August 2020, the patient experienced sudden chills, shivering, and hyperpyrexia. Sputum culture revealed *Candida albicans*, and chest computed tomography (CT) showed multiple nodules in the lungs (Figures 2A,B). The 1,3-β-D glucan test (G test) and galactomannan antigen detection test (GM test) were negative. Due to poor coagulation, tracheoscopy was not performed. Pulmonary candidiasis was diagnosed, and the patient’s fever persisted after a week of treatment with fluconazole 400 mg/d ivgtt. A repeat sputum culture on 14 August 2020 revealed *Aspergillus niger*, resulting in a diagnosis of invasive pulmonary aspergillosis (IPA). Fluconazole was changed to voriconazole (loading dose on 18 August 2020 of 400 mg q12 h ivgtt, and maintenance dose of 200 mg q12 h ivgtt was started on D2. The regimen was adjusted according to the clinical situation and therapeutic drug monitoring (TDM) results), and his condition improved.

His chest CT showed resolution of the lung lesions after 85 days of treatment, and voriconazole was discontinued on 10 November 2020. On 31 May 2021, laboratory studies revealed TBil, 11.9 μmol/L; ALT, 52 U/L; AST, 40 U/L; ALP, 204 U/L; and γ-GT, 93 U/L. A repeat liver biopsy after 24 weeks of treatment was consistent with autoimmune-like phenomenon liver failure (G3 S3) (Figures 1B,C). A diagnosis was made according to the Roussel Uclaf Causality Assessment Method (RUCAM) (Danan and Benichou, 1993), and a probable diagnosis of autoimmune hepatitis was made based on 2008 International Autoimmune Hepatitis Group’s (IAIHG) criteria (Hennes et al., 2008): (1) DILI, cholestatic, acute, and RUCAM score calculated as 10 (highly likely); (2) IAIHG score 4, combined with liver pathology, the diagnosis of the teenager is drug-induced liver injury with autoimmune-like phenomenon (AL-DILI), drug-induced autoimmune hepatitis (DI-AIH) is not excluded.

### Case 2

A 17-year-old girl (weight: 53 kg; height: 160 cm; BMI: 20.70 kg/m^2^) was admitted to our hospital on 6 July 2020 complaining of dysphoria, fatigue, vomiting, and yellow eyes which began 9 days before admission. She had been treated with a Chinese patent medicine, QubaiBabuqi tablets (4 tablets t.i.d. for more than 3 months) for vitiligo before the onset of the symptoms. Physical examination showed severe jaundice of the skin and sclera, and localized whitish patches on her face. No cardiopulmonary abnormalities were noted. Laboratory studies on July 7 revealed: TBil, 439.60 μmol/L; DBil, 325.70 μmol/L; ALT, 1174 U/L; AST, 505 U/L; γ-GT, 131 U/L; ALP, 166 U/L; PT, 44.5 s; prothrombin time activity (PTA), 16.00%; INR, 4.84; and blood ammonia, 258 μmol/L. A diagnosis of drug-induced acute liver failure was considered.

**FIGURE 3:**
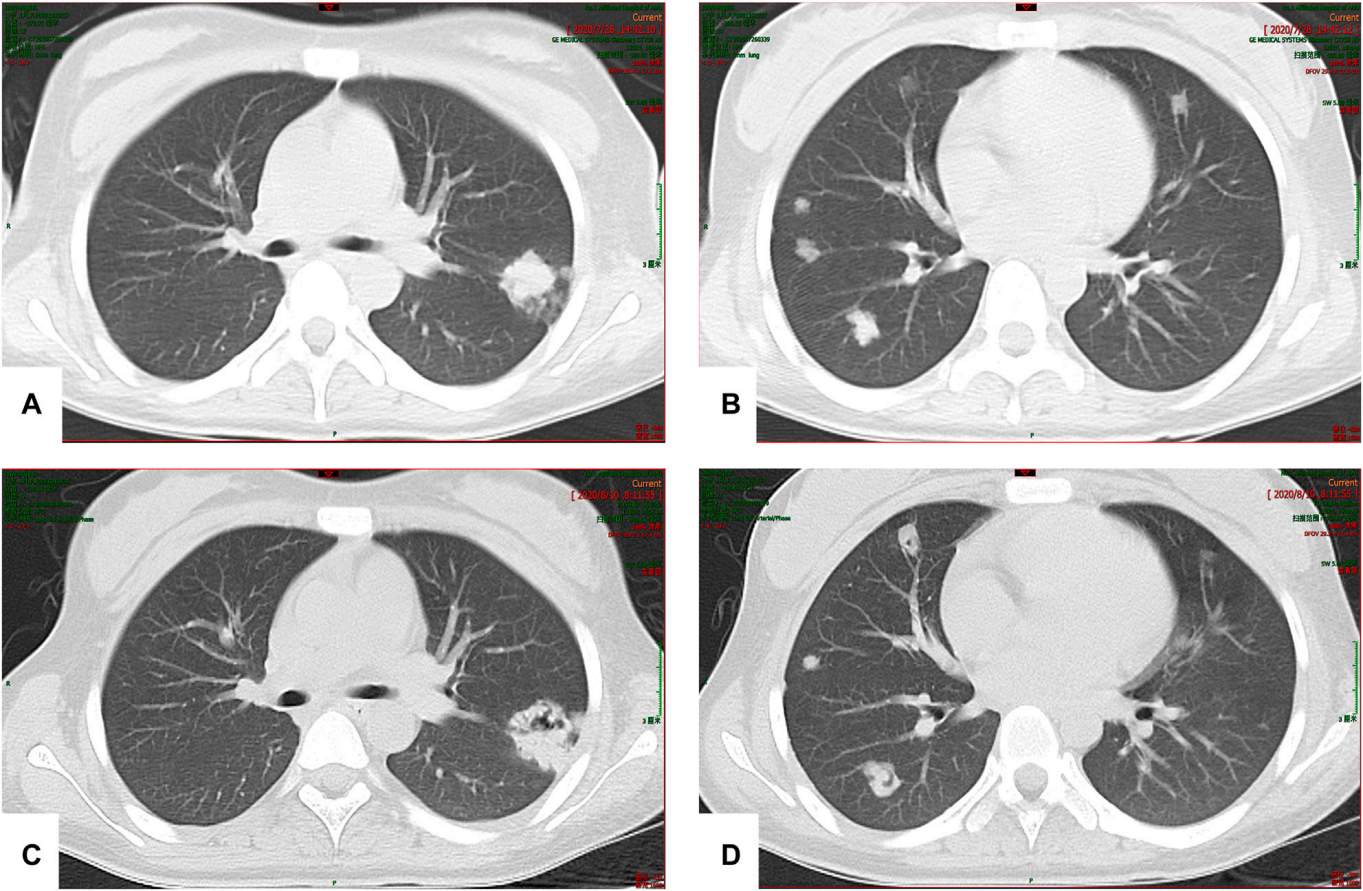
**(A,B)** Multiple nodular high-density shadows were observed in both lungs; the larger one was located in the upper lobe of the left lung (20 × 24 mm). **(C,D)** After 2 weeks of treatment, multiple nodular high-density shadows were observed in both lungs; the larger one was 29.4 × 28.4 mm in size, and voids were present in some lesions.

**FIGURE 4:**
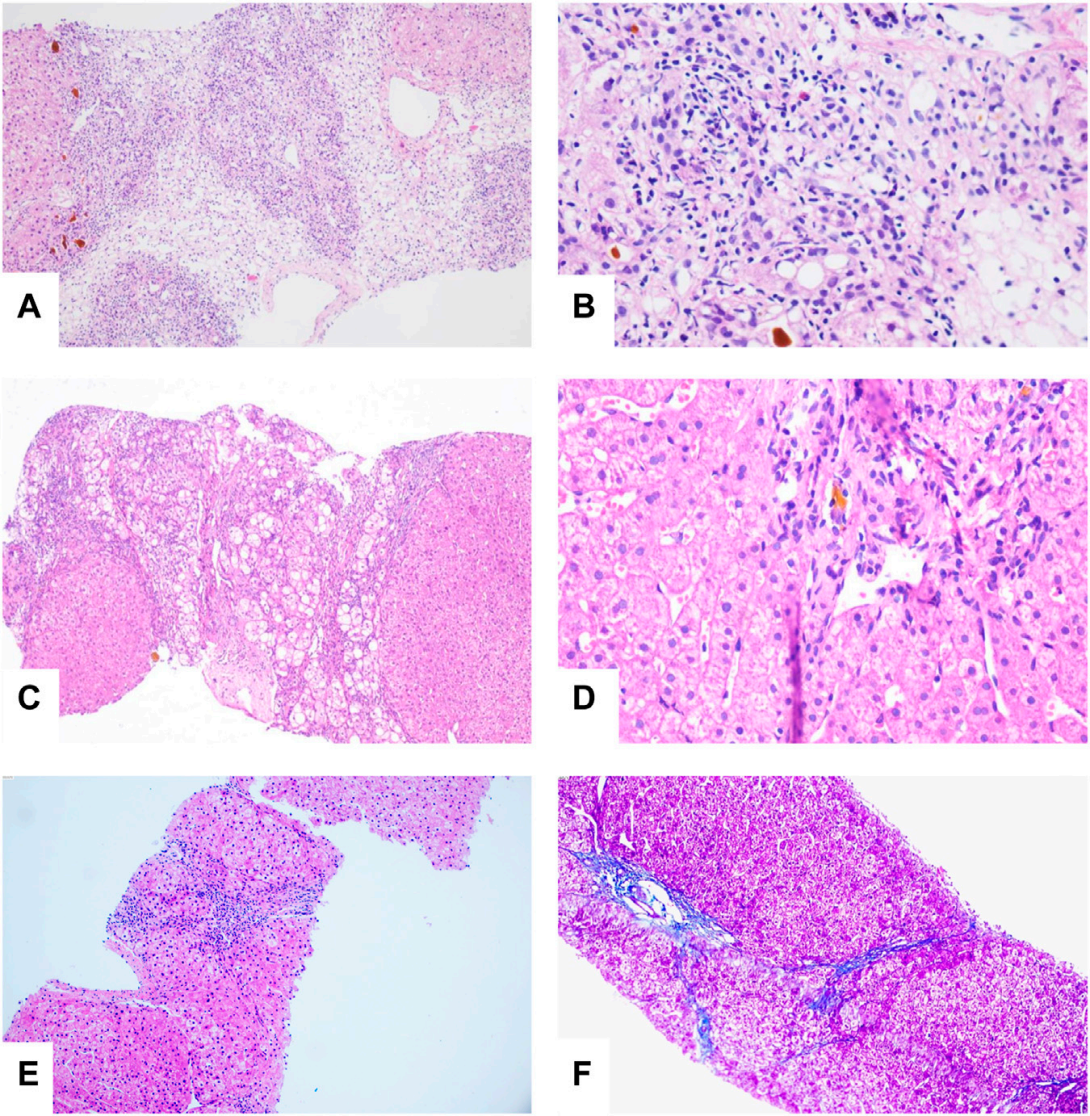
**(A)** Bridging or submass necrosis of hepatocytes and residue liver cells were arranged in a pseudolobule-like pattern, some of which had macrovesicular steatosis (<10%), and moderate to severe interfacial inflammation were noted (HE, original magnification × 40). **(B)** The portal duct area was obviously enlarged, interlobular bile duct hyperplasia was present, and portions of the bile ducts were slightly dilated with bile thrombosis. Infiltration of numerous mixed inflammatory cells into the interstitium, and fibrous tissue hyperplasia accompanied with partial pseudolobule formation was also noted (HE, original magnification × 100). **(C)** “Rose garland” formation (HE, original magnification × 40). **(D)** Lymphocytes “penetrate” (HE, original magnification × 200). **(E,F)** Slight edema and degeneration in hepatocytes, scattered with spotty necrosis. Infiltration of a small number of mixed inflammatory cells into the interstitium and fibrous tissue (HE, original magnification × 40).

The patient developed hepatic encephalopathy and frequent vomiting on day 4, and she was placed on the liver transplant waiting list. She was treated with dexamethasone (10 mg q.d. i.v., gradually decreased after 3 days), liver protection, and NBAL (from July 7 to July 20, once every 3–4 days), and her liver function rapidly improved. After 10 days in a comatose state, she regained consciousness. She continued to have an intermittent low-grade fever on 24 July 2020, and no cough or other symptoms. Her TBil level had increased to 388 μmol/L, and the white blood cell (WBC) count was 12.13 × 10^9^/L with 83.50% neutrophils. Her chest CT scan showed multiple nodules in both lungs on July 26 (Figures 3A,B), and IFI was diagnosed. A loading dose of caspofungin of 70 mg q.d. ivgtt was given on the first day, and then it was continued at a dose of 35 mg/d ivgtt for 23 days. However, a repeat chest CT showed an increase in the number of lung lesions and the presence of an air crescent sign (Figures 3C,D). Bronchoalveolar lavage fluid (BALF), G test, GM test, and next-generation sequencing (NGS) were all negative; however, based on her history and chest CT findings, a diagnosis of IPA was highly likely. Caspofungin was changed to voriconazole (loading dose on D1 of 6 mg/kg daily q12 h ivgtt, maintenance dose of 4 mg/kg q12 h orally was started on D2) on 19 August 2020, and her liver function gradually improved. A liver biopsy on 25 August 2020 was consistent with DILI (G 3–4 S 3–4) (Figures 4A,B). She was discharged on oral voriconazole (250 mg, q12 h), methylprednisolone (4 mg, q.d.), and other medications. A second liver biopsy after 10 weeks revealed an autoimmune-like phenomena hepatitis (G 1–2 S 2–3) (Figures 4C,D). A liver biopsy was performed, and ink staining, periodic acid–Schiff staining, hexamine silver staining, acid-fast staining, and NGS of liver tissue were negative. A diagnosis of DILI was made according to the RUCAM and 2008 IAIHG criteria: (1) DILI, hepatocellular damage-type, acute, RUCAM score calculated as 9 (highly likely); (2) IAIHG score 4, the diagnosis of DILI with autoimmune-like phenomena.

In order to confirm the diagnosis and efficacy, we performed a third liver biopsy a year later (12 August 2021). The liver histopathology indicated chronic hepatitis (G 1 S 2) (Figures 4E,F), which was significantly improved compared with the previous two. No autoimmune-like phenomena were observed. Therefore, we have discontinued glucocorticoids on 18 August 2021. After 406 days of voriconazole treatment, the pulmonary nodules were completely absorbed.

In both patients, serological studies for hepatitis A virus (HAV), hepatitis B virus (HBV), hepatitis C virus (HCV), hepatitis E virus (HEV), and human immunodeficiency virus (HIV) were negative. Laboratory studies for cytomegalovirus nucleic acid (CMV-DNA), Epstein–Barr virus nucleic acid (EBV-DNA), and herpes simplex virus (HSV) IgM antibody were negative. Serum copper, ceruloplasmin, iron, and ferritin levels were normal. Tests of thyroid function and for genetic metabolic liver diseases were normal. Ultrasound scans of the hepatobiliary system did not reveal any abnormalities. MRI plain scan, enhanced MRI scan, magnetic resonance cholangiopancreatography (MRCP), and diffusion-weighted imaging (DWI) of the liver indicated a diffuse liver injury, and no bile duct dilatation was observed on MRCP. Antinuclear antibody (ANA), anti-smooth muscle antibody (ASMA), anti-liver and kidney microsomal type 1 antibody (LKM1), anti-hepatocyte cytoplasmic type 1 antibody (LC1), anti-soluble liver antigen antibody (SLA) and immunoglobulin G (IgG), γ-globulin, G test, GM test, blood cultures, and *Cryptococcus* capsular antigen tests were performed several times during the course of each patient’s disease, and all tests were negative. In both patients, after voriconazole reached a steady state, that is, in the morning of the 4th day or after adjusting the dose for 5 days, in the morning of the 6^th^ day, 30–60 min before fasting medication, the contralateral venous blood was collected to detect the concentration of voriconazole valley, and the therapeutic drug monitoring (TDM) of voriconazole was carried out. The regimen was adjusted according to the clinical situation and voriconazole TDM results (Table 1). The liver pathological examination results were read by two pathologists.

**TABLE 1:** Usage of voriconazole was adjusted according to the clinical situation and therapeutic drug monitoring (TDM) results.

Case	Date	TBil ( μmol/L)	ALT(U/L)	Symptoms	Chest CT	Sputum culture	TDM of voriconazole (μg/ml)	Voriconazole dose	Voriconazole usage
1	7-Aug-2020	—	—	Sudden chills, shivering, and hyperpyrexia	Multiple nodules in the lungs	—	—	—	—
1	14-Aug-2020	258.2	54	Normal temperature	—	*Aspergillus niger*	—	—	—
1	17-Aug-2020	—	—	Asymptomatic	—	—	—	400 mg q 12 h	ivgtt
1	18-Aug-2020	—	—	Asymptomatic	—	—	—	200 mg q 12 h	ivgtt
1	22-Aug-2020	175.7	54	Asymptomatic	—	—	—	200 mg q 12 h	ivgtt
1	26-Aug-2020	121.3	65	Asymptomatic	—	—	5.83	150 mg q 12 h	Oral
1	1-Sep-2020	87.1	67	Asymptomatic	—	—	5.84	150 mg q 12 h	Oral
1	4-Sep-2020	—	—	Asymptomatic	—	—	—	100 mg q 12 h	Oral
1	7-Sep-2020	65.5	65	Asymptomatic	—	—	4.19	100 mg q 12 h	Oral
1	16-Sep-2020	65.9	86	Asymptomatic	Multiple nodules in both lungs were absorbed	—	3.5	100 mg q 12 h	Oral
1	23-Sep-2020	53.2	86	Asymptomatic	—	—	—	100 mg q 12 h	Oral
1	9-Oct-2020	36.7	104	Asymptomatic	—	—	1.66	150 mg q 12 h	Oral
1	23-Oct-2020	31	98	Asymptomatic	The absorption of nodules was not obvious	—	1.46	150 mg q 12 h	Oral
1	9-Nov-2020	25.4	103	Asymptomatic	—	—	1.76	150 mg q 12 h	Oral
1	10-Nov-2020	—	—	Asymptomatic	—	—	—	Drug withdrawal	—
2	26-Jul-2020	—	—	Intermittent low-grade fever	Multiple nodules in both lungs	—	—	—	—
2	10-Aug-2020	131	109	Intermittent low-grade fever	Increased nodules in both lungs and the presence of an air crescent sign	—	—	—	—
2	17-Aug-2020	80	52	Normal temperature	—	—	—	—	—
2	19-Aug-2020			Asymptomatic	—	—	—	300 mg q 12 h	ivgtt
2	20-Aug-2020			Asymptomatic	—	—	—	200 mg q 12 h	Oral
2	24-Aug-2020	64	37	Asymptomatic	—	—	6.16	150 mg q 12 h	Oral
2	4-Sep-2020	35.6	34	Asymptomatic	—	—	4.43	150 mg q 12 h	Oral
2	25-Sep-2020	22.5	18	Asymptomatic	—	—	1.95	150 mg q 12 h	Oral
2	13-Oct-2020	20.4	20	Asymptomatic	—	—	2	150 mg q 12 h	Oral
2	2-Nov-2020	11.6	14	Asymptomatic	Nodules are enlarged in both lungs	—	2.51	200 mg q 12 h	ivgtt
2	6-Nov-2020	14.4	14	Asymptomatic	—	—	—	200 mg q 12 h	ivgtt
2	13-Nov-2020	16.5	12	Asymptomatic	—	—	4.65	200 mg q 12 h	ivgtt
2	16-Nov-2020	17.4	13	Asymptomatic	Nodules were absorbed	—	1.27	200 mg q 12 h	ivgtt
2	20-Nov-2020	—	—	Asymptomatic	—	—	1.45	200 mg q 12 h	Oral
2	23-Nov-2020	—	—	Asymptomatic	—	—	2.15	200 mg q 12 h	Oral
2	24-Nov-2020	8.7	14	Asymptomatic	—	—	—	200 mg q 12 h	Oral
2	2-Dec-2020	18.1	17	Asymptomatic	The absorption of nodules was not obvious	—	3.81	250 mg q 12 h	Oral
2	18-Dec-2020	13.9	14	Asymptomatic	—	—	2.85	250 mg q 12 h	Oral
2	11-Jan-2021	14.2	16	Asymptomatic	—	—	3.88	250 mg q 12 h	Oral
2	17-Feb-2021	8.5	17	Asymptomatic	—	—	3.24	250 mg q 12 h	Oral
2	19-Mar-2021	8.8	13	Asymptomatic	Nodules were absorbed	—	4.09	200 mg q 12 h	Oral
2	30-Apr-2021	11.6	15	Asymptomatic	—	—	2.78	200 mg q 12 h	Oral
2	4-Jun-2021	9	14	Asymptomatic	—	—	2.52	200 mg q 12 h	Oral
2	28-Sep-2021	—	—	Asymptomatic	—	—	—	Drug withdrawal	—

Note: voriconazole plasma concentration (normal range 1.5–5.5 μg/ml).

## Discussion

DILI is caused by almost 1,000 medications, including herbal supplements and dietary supplements (Amin et al., 2015). Chinese patent medicines are traditional Chinese medicine products, some of which contain Western medicine ingredients such as acetaminophen, chlorpheniramine maleate, and other Western medicines. In a retrospective study of pediatric DILI in China, Chinese herbal medicine and combined drugs each accounted for 21.7% of cases, while Western medicines accounted for 56% of cases (Zhu et al., 2015).

Acetaminophen-related ALF and idiosyncratic drug-induced liver injury-related ALF account for more than 50% of all acute liver failure cases in the United States and may progress rapidly to death within 72 h (Chayanupatkul and Schiano, 2020). Case 1 developed a rash after taking an acetaminophen-containing Chinese patent medicine, developed severe liver function impairment 1 week later, and progressed to subacute liver failure 2 months later. Qubaibabuqi tablets were taken by Case 2, which contains the Chinese herb *Psoralea* that is known to induce oxidative stress that may cause DILI (Xu et al., 2021). The patient progressed to ALF and hepatic encephalopathy after using the medicine for more than 3 months.

Liver biopsy remains an important tool in the diagnosis of liver diseases, and interface hepatitis and lymphocytic/lymphoplamocytic plasma cell and hepatic rosette formation are regarded as pathological characteristics of typical autoimmune hepatitis. Both penetration and rosettes are significantly associated with autoimmune hepatitis and are considered to be more significant than interface hepatitis or plasma cell infiltration (de Boer et al., 2015). The penetration phenomenon refers to the entry of lymphocytes into the cytoplasm of hepatocytes, while the rosette is a pseudoglandular structure formed by several hydromorphic hepatocytes surrounded by lymphocytes. Studies showed that 65% of patients with autoimmune hepatitis present with penetration, whereas only 33% of patients with rosettes. However, these features are also seen in acute and chronic liver diseases of different etiologies (Balitzer et al., 2017).

Diagnoses of autoimmune-like phenomenon DILI and drug-induced autoimmune hepatitis cannot be excluded in the first patient presented herein based on elevated levels of serum transaminase, the histopathological findings of AIH presentation, the effective treatment of glucocorticoid, normal serum GLO, γ-globulin, IgG, ANA, SMA, and LKM1 levels. Two studies proposed that histological features of autoimmune-like phenomenon DILI and drug-induced autoimmune hepatitis are similar to those found in classical autoimmune hepatitis, and there is no advanced hepatic fibrosis or cirrhosis in most cases (Lewis, 2011; Flamm et al., 2017). Different degrees of liver fibrosis were found in the 2 cases presented herein, which may be related to post-necrotic cirrhosis (Child–Pugh class C) caused by the previous occurrence of hepatocyte necrosis, or significant fibrosis, present in the early stage of the disease due to their critical condition. Although liver biopsy is the gold standard for the diagnosis of liver diseases, further studies are needed to develop methods to distinguish the three conditions. Currently, there is no standard for diagnosis of autoimmune hepatitis in teenagers who are both autoantibody- and IgG-negative. The liver pathology of both patients showed an autoimmune-like phenomenon DILI, and the liver histology improved after glucocorticoid therapy. The course of glucocorticoid therapy in case 1 was shorter than that of drug-induced autoimmune hepatitis (generally more than 2 years). Case 2 was still using glucocorticoid, but her liver function was normal. She was in the process of gradual reduction and was ready to stop using glucocorticoids after completing the third liver biopsy. Therefore, autoimmune-like phenomenon DILI may have a better prognosis and a shorter course of glucocorticoid therapy than drug-induced autoimmune hepatitis.

Both patients presented herein had severe liver injuries and hepatic encephalopathy, with a PT > 15 s and INR >1.5, which could not be corrected by vitamin K. The diagnoses of drug-induced subacute liver failure and drug-induced acute liver failure were made based on the 2017 Hepatology Society of European’s Clinical Practice Guideline for the management of acute (fulminant) liver failure (European Association for the Study of the Liver et al., 2017). For patients with severe acute liver injury, liver transplantation should be performed immediately if the condition does not improve within 7 days. However, due to the shortage of donor livers in China and the high cost of the surgical procedure, only a few patients receive liver transplantation in time. The American Gastroenterological Association Institute (AGA) recommends that extracorporeal liver support systems be used only for clinical testing in order to allow the body to recover and avoid liver transplantation; this strategy can be lifesaving for patients awaiting liver transplantation (Flamm et al., 2017).

The condition of patient 1 did not improve after methylprednisolone, MMF, and intravenous gamma globulin, but liver function did improve after 5 treatments with NBAL. Even if case 2 developed HE after NBAL treatment, PT decreased within a short span of time and liver function improved. Both of the patients survived without liver transplantation, suggesting that NBAL treatments may be beneficial for patients with liver failure. The successful outcomes of the 2 patients were related to young age, prompt diagnosis, rational use of glucocorticoids, good liver regeneration ability, timely NBAL treatments, and rapid treatment of secondary IFI. However, treatment with glucocorticoids can cause sepsis and increase the fatality rate (European Association for the Study of the Liver et al., 2017). Two patients used glucocorticoids for a long time, which led to the immunosuppressive state, resulting in the high incidence of IFI. Notably, the use of an indwelling catheter increases the risk of catheter-related bloodstream infections and fungal infections; thus, we should rigorously be aware of the indications, timing, and course of glucocorticoid treatment for patients with liver failure.

The numbers of patients with invasive fungal infections have been increasing yearly, and these infections can cause tissue damage, organ dysfunction, and a severe inflammatory reaction. *Aspergillus niger* was cultured in the sputum of patient 1, but no studies were positive for fungi in patient 2. Nevertheless, IPA was clearly diagnosed based on medical history, treatments and outcomes, and lung imaging changes. A clinical study retrospectively reviewed and confirmed probable cases of invasive aspergillosis in children from six major medical centers. The most common site of infection was the lungs (59%), and nodules were most commonly diagnosed on imaging (34.6%). An air crescent sign was seen in only 2.2% of the children, a halo sign in 11%, and cavitation in 24.5%. Furthermore, chest CT of children with confirmed IPA usually has only non-specific signs, rather than halo signs, air crescent formation, or voids seen in adults (Donnelly et al., 2020). The 2 patients presented herein both exhibited typical pulmonary imaging manifestations of IPA, such as multiple pulmonary nodules and halo sign in the early stage, and air crescent sign and void in the later stage, the typical signs allowed for an early diagnosis and treatment.

IPA is associated with a high mortality rate, and the 28-day mortality rate of liver failure combined with an invasive fungal infection is 56%, and the 90-day mortality rate is 71% (Fernández et al., 2018). IFI is the main cause of death in patients with acute liver failure, and the G test and GM test are useful markers to guide antifungal therapy in patients (Verma et al., 2019). In our report, tests of bronchoalveolar lavage fluid and multiple peripheral blood G tests and GM tests were negative in the second teenager, suggesting that the sensitivity of the G test and GM test is limited and that more reliable serological markers are needed to establish an early diagnosis.

Voriconazole is a first-line agent for the treatment of invasive *Aspergillus* infection in teenagers with its main elimination via liver metabolism. Impaired hepatic function may delay clearance; it may lead to a disproportionate increase in blood concentration and a significantly reduced clearance of voriconazole. Voriconazole concentrations and dosage vary significantly in patients. Pediatric patients require higher doses to achieve an exposure similar to adults (Mantadakis et al., 2019); the reason in part is due to the CYP2C19 allele variation. Voriconazole concentrations in plasma was decreased in rapid metabolizer of CYP2C19, resulting in a delayed of the target plasma concentration, whereas, those with poor metabolism an increased risk of the trough concentration and adverse drug events increased. Genetic polymorphism analysis of CYP2C19 in Chinese Han populations had the greatest effect on voriconazole, of them, CYP2C19* 2 and CYP2C19* 3 are the major mutant alleles (Moriyama et al., 2017); hence, gene polymorphism detection should be conducted before the use of voriconazole. In a retrospective study of 78 patients with Child–Pugh grade B and C cirrhosis who were treated with voriconazole showed that 62.79 and 28.36% of patients with maintenance 200 mg q12 h, respectively, with a Cmin <5 mg/L. The probability of voriconazole-related adverse events was 87.5% within 7 days (Wang et al., 2018). During the treatment of voriconazole, the visual impairment, rash, muscle weakness, and other symptoms occurred in case 1, but when the blood concentration of voriconazole was reduced to the normal range, the visual impairment and muscle weakness were relieved and the rash resolved after the drug withdrawal. No voriconazole-related adverse events were reported in case 2. In general, voriconazole was safe in these two cases, and no voriconazole-related DILI was found. Among them, case 2 received the oral voriconazole dosage (250 mg q12 h) greater than the conventional dose, which may be related to the fast hepatic blood flow velocity, fast metabolism, and a more obvious first-pass effect of the drug in teenagers. Physiological factors such as age, sex, age, and body weight; CYP2C19 gene polymorphism; and pathological factors such as albumin, CRP, liver and kidney function, and the interaction between drugs and other drug combinations can all affect the plasma concentration of voriconazole, and individual regimen should be formulated during treatment.

DILI in teenagers caused by Chinese patent medicines is severe and can rapidly progress to liver failure. Some of the patients can benefit from comprehensive medical therapy including glucocorticoids and NBAL treatments and avoid the need for liver transplantation. It is important to point out that the treatment of glucocorticoid in the early stage did not prevent the progress of the disease. Maybe majority of autoimmune-like phenomena DILI can be successfully treated by glucocorticoid. However, secondary IFI may result in a poor response to glucocorticoid therapy. Hence, indications, treatments, and timings should be carefully planned, and patients should be closely monitored for adverse reactions. The diagnosis of autoimmune-like phenomena DILI was not only based on the changes of liver pathology before and after treatment but also combined with the response after glucocorticoids treatment and whether it recurred after drug withdrawal.

## Conclusion

Voriconazole has a good curative effect and safety profile in severe liver damage (Child–Pugh class C) in teenagers. TDM should be monitored, and attention should be given to the occurrence of adverse drug reactions. Drug-induced autoimmune-like phenomena could be improved by glucocorticoid therapy. Glucocorticoid is a double-edged sword. We need to be vigilant against fungal infection in the DILI treatment with glucocorticoid. The sensitivity of serological markers such as the G test and the GM test for the diagnosis of an IFI is not high, and there are many influencing factors. As such, further study is needed to establish markers that can make an early diagnosis of IFI.

## Data Availability

The original contributions presented in the study are included in the article/Supplementary Material, further inquiries can be directed to the corresponding author.
